# Supplementary material for: Utilizing Headspace–Gas Chromatography–Ion Mobility Spectroscopy Technology to Establish the Volatile Chemical Component Fingerprint Profiles of Schisandra chinensis Processed by Different Preparation Methods and to Perform Differential Analysis of Their Components
Source: Molecules. 2024 Dec 13;29(24):5883. doi: 10.3390/molecules29245883 (PMC11677488; doi:10.3390/molecules29245883)
Supplement: Supplementary file 1 [file molecules-29-05883-s001.zip › Basic parameters of the experimental equipment.pdf]

Supplementary Materials

Basic parameters of the experimental equipment

Gas Chromatography-Ion Mobility Spectrometry (FlavourSpec®) Technical Parameters：

Main Unit Output Voltage: 24V DC

Working Environment Temperature: 22°C

Working Environment Humidity: 30%

Carrier Gas: High-purity Nitrogen (99.999%)

Ion Mobility Spectrometry Technical Parameters:

Detector: IMS

Ionization Source: Radioactive Tritium Source (H^3)

Ionization Source Radiation Type: Beta (β) Radiation

Ionization Source Radiation Energy: 5.68-18.7 KeV

Ion Mobility Spectrometry Resolution (Peak Capacity): > 40

Drift Tube Length: 98mm

Drift Tube Voltage: 5000V
